# Astute exploration of collective mental health events among the residents of elderly care homes

**DOI:** 10.1016/j.heliyon.2023.e18117

**Published:** 2023-07-08

**Authors:** Nasrin Akter, Bilkis Banu, Sujana Haque Chowdhury, Kazi Rakibul Islam, Tahsin Tasneem Tabassum, Sarder Mahmud Hossain

**Affiliations:** a Department of Public Health, Northern University Bangladesh, Dhaka, Bangladesh; b Department of Community Medicine, East-West Medical College, Dhaka, Bangladesh

**Keywords:** Collective mental health events, Exploration, Elderly care homes

## Abstract

**Background & objective:**

Developing countries are presently witnessing a great burden of rapid aging followed by losing the social values of older adults due to age-related cognitive impairment as well as rising depression levels. This study was designed to assess the cognitive impairment and depression status combinedly among older adults in elderly care homes.

**Methods:**

It was a cross-sectional survey among randomly selected 200 older adults aged between 60 and 80 years residing in some old homes in Dhaka district, Bangladesh. Data were collected through face-to-face interviews while Cognitive function and level of depression were assessed by applying the Standardized Mini-Mental State Examination (MMSE) and Geriatric Depression Scale (GDS).

**Results:**

Among all the respondents, the majority (81.5%) were staying in old homes for 1–5 years. The majority (91.0%) had difficulties with vision, 40.7% had difficulties with hearing and 19.6% had difficulties moving around. The MMSE test revealed that 43% had moderate cognitive impairment, 36% had mild and 19.5% were found normal while more than half (56%) had severe depression. Significant cognitive impairment was found among the illiterate respondents who did not have any family care support. In addition, higher-educated respondents were found to have more severe depression (OR/p = 6.33/<0.01; 95% CI: 2.36–16.96). Furthermore, severely depressed respondents had more severe cognitive impairment (COR/p = 3.83/0.01; 95% CI: 1.66–8.83). Functional disabilities were also a greater concern for cognitive impairment and depression.

**Conclusion:**

An increasing number of old home residents are suffering from significant mental disorders while there is no mental health support in the elderly care homes in Bangladesh. Finally, there is a great need to develop packages and programs of mental health care for senior citizens and their caregivers residing in old homes, which can be scaled up across the country’s mixed healthcare delivery system.

## Highlights

• Crucial determinants for both cognitive impairment and depression are assessed combinedly among elderly care home residents.
• Such empirical exploration lacks in this vulnerable group which is inevitable to combat the public health burden.
• The unique outcome may promote psychological stress management aids and facilitate healthcare support for this specific group.

## 1 Introduction

Population aging is a demographically inevitable and complex process. Around 8% of the total population in Bangladesh was reported to be aged over 60 years as of 2019 and this proportion is expected to soar to 21.9% in 2050 [1]. Owing to increased overseas migration of the young generation, improved female employment rates, globalization, and family support systems have reduced leading to the need for alternative care facilities such as old homes. These paid or charitable homes are one of the places where the geriatric population is institutionalized [2]. Many older adults suffer from age-associated mental disorders such as cognitive decline or cognitive impairment and depression [3].

Cognitive disorders can be associated with a decline in cognitive abilities like attention, memory, language, orientation, performance, judgment, and problem-solving skills [4]. International studies have shown that about 47.5% of older adults have a moderate cognitive disorder, 30% have a small disorder 5% have a severe disorder [5]. The prevalence of cognitive disorders in Swedish, German, Portugal, and American older people is 4.5%, 7.7%, 9.6%, and 4.9, respectively [[6], [7], [8]]. A study in southern urban India revealed that 42.7% of older residents of care homes had cognitive impairment, whereas, among the community dwellers, 21.9% of the older adults had it [2]. On the contrary, in Bangladesh, by the year 2021, 21.6% of the older adults suffer from mild to moderate cognitive impairment whereas 6.3% suffer from a severe impairment, while previously dementia and mild cognitive impairment were found prominent among the older adults [9,10]. This variability may be due to the types of study and different diagnostic criteria used in the study. However, none of these explorations were among the specific target group (residents of old homes) who are considered in this study. This group is much more vulnerable rather than the previously explored group from the household setting as they are found to be deprived of family care support and the health care facility in old homes of Bangladesh is pretty poor. The crude prevalence of mild cognitive impairment is higher in the Indian subcontinent rather than the other areas of the world like America and China [11]. It is also associated with higher mortality especially observed among the elderly population residing in care homes [12,13].

Depression is also one of the main reasons for disability among older adults which has accentuated the risk of mortality and morbidity [14,15]. Loss of a spouse, being away from children, usage of multiple drugs, and having chronic illnesses with increasing age along with the loss of cognitive power have made senior adults vulnerable to depression [16]. The prevalence of depression among older adults has been estimated to be between 8% and 15% which reaches 30% in the older residents of nursing homes [17]. The senior adults in old homes were found to be more depressed in comparison to those of the community in India [18]. These studies, based on selected populations and sometimes failed to demonstrate the scenario of the general population. In Bangladesh, a majority of older adults in old homes or communities are found to develop the symptoms of depression. A good number of studies were conducted in Bangladesh to explore geriatric health issues including depression which are often ignored, although they are a growing segment of society [[19], [20], [21], [22]]. The empirical studies lack the combined exploration of depression and cognitive impairment or dementia among the older adults especially those residing in old homes.

Owing to major psychological problems like depression and cognitive impairment, the aging of the population is deemed to be one of the most dominant causes of negative health consequences including a decrease in the quality of life in recent decades [17,23,24]. Evidence from a community-based cohort study in America shows the decline in daily living activities is significantly associated with cognitive impairment as well as depression [25]. Such combined exploration is inevitable in a country like Bangladesh where the prevalence of cognitive impairment and depression is comparatively higher and reduced resources or support for senior adults in a negligible manner. In addition, the combined exploration including an inner association between cognitive impairment and depression is not explored yet among the Bangladeshi older adult group. Therefore, It is the greatest challenge is to prevent disabilities due to the double burden of cognitive impairment and depression as well as improve the quality of life of older adults residing in elderly care homes [26].

Reflecting on intellectual contributions toward the health and well-being of older adults, it is intelligible that exploration of the interaction of cognitive impairment and depression status including the responsible determinants is pivotal for the well-being of older adults living in elder care facilities. Therefore, the current study aims to explore the combined distribution of cognitive impairment and depression including the internal association posing a double burden among the respondents in a single snapshot, and simultaneously to reveal the triggering factors of such health issues among older adults from old homes.

The impactful outcome of this study might facilitate future endeavors in screening and treatment of both cognitive impairment and depression among senior citizens living in old homes through scaling up strategies in healthcare facilities. In addition, guidance regarding social awareness and creating opportunities like active participation of older adults may be executed from the effective recommendations of this study.

## 2 Method

### 2.1 Study design

This descriptive type of cross-sectional study was conducted based on a quantitative research design. The semi-structured data were collected during August 2021 to portray cognitive impairment and depression including its associated factors among residents of elderly care homes in Bangladesh.

### 2.2 Study participants, sample size, and sampling

A total of 200 respondents were enrolled in this study who were aged between 60 and 80 years and residents of the elderly care home of Dhaka district of Bangladesh. Considering the multi-variation of socio-cultural context, this study was conducted in four renowned elderly care homes i.e. Probin Nibash, Dhaka; Old Rehabilitation Centre, Gazipur; Apon Nibash Old Age Home, Dhaka; Child and Old Age Care, Mirpur, Dhaka. Initially, it was assumed that a potential standard sample size of 245 would be taken by using the formula “n = ‘Z^2^pq/d^2^” where Z (standard normal deviate) is considered as 1.96 at 95% CI; p (proportion of cognitive impairment of older adult people) was considered as 0.20 [27] and margin of error was considered as 0.05. However, the final sample size was directed to 200 where the presumed response rate was considered 82% according to their ability to respond to the interviewer-administered data collection instrument as they were aged enough leads to their distorted usual physical and mental health status mostly. In addition, still less number of old age homes in Bangladesh against a huge number of growing older adults [28]. Therefore, 200 samples are considered adequate to yield significant statistical analysis used in this study as well as reveal a valid outcome. Among the total respondents, 50 were enrolled from each elderly care home following an equal proportion according to the selected number of old homes. The lists of older residents were collected from the authority of elderly care homes and then every 50 respondents for each old home were selected from the list following the systematic random sampling technique. Older adults were included as study participants who gave consent to attend this study and were observed as physically and mentally sound.

### 2.3 Data collection

Data were gathered through face-to-face interview method by the recruited data collectors. A pre-tested and semi-structured questionnaire was used to collect the information. Due to the spread of the COVID-19 pandemic, data were collected by the maintenance of social distancing and using personal protective equipment (PPE) as a preventive strategy. The interview took only 10–15 min to complete. The survey was administered in the local Bengali language with the utmost support of the elderly care home authority. Cognitive function was assessed by applying a standardized Mini-Mental State Examination (MMSE) in Bangla [29]. Depression was assessed with the Geriatric Depression Scale (GDS) [30].

### 2.4 Ethical considerations

This study was approved by the Ethical Review Committee of Northern University Bangladesh (NUB) (NUB/DPH/EC/2021/08-a) and conformed to the Declaration of Helsinki. Participation of the respondents was anonymous and voluntary. In Bangladesh, old-home residents are getting physical and mental health services from the authority according to the structural concept of each old home. To collect data, the sampling frame was constructed through the recommended list of physically and mentally stable older adults provided by the authority. This selection was done according to their medical records and caregivers' recommendations. Written informed consent was taken from the respondents including their authority/caregivers at the beginning of the survey and participants could withdraw from the survey at any time. For respondents who were not able to write their names due to disabilities, we took thumbprints of them.

### 2.5 Questionnaire design

The questionnaire was pre-validated by two independent reviewers and pre-tested among 10 respondents. The responses from the pre-test were used to improve the quality of the questionnaire. The questionnaire comprised several segments: (i) Assessment of cognitive impairment status among older adults; (ii) Identification of level of depression among older persons; (iii) Demography of the older persons: age, gender, education, past occupation; (iv) Health status: comorbidities, physical disability for regular works; (v) Reasons for staying at the old home.

### 2.6 Data analysis

The quality of data was checked and analyzed employing the Statistical Package for the Social Sciences (SPSS) software version 21. Study characteristics were subjected to descriptive statistics (frequency and proportions) to summarize the obtained data. To categorize the data of age the cut-off value was decided according to the mean age value of the respondents. MMSE was scored ranging from 0 to 30. Lower scores (0–9) indicated increasing severity of cognitive impairments in the domains of orientation, memory, attention, and executive functions. Subjects with moderate cognitive impairment had scores between 10 and 21. The scores of 21–24 were considered mild and 25 to 30 were normal [29]. To analyze the depression status the geriatric depression scale was used where out of the 15 items, 10 scored as the presence of depression when answered positively, and the rest (question numbers 1, 5, 7, 11, 13) scored as depression when answered negatively. Scores of 0–4 were considered normal; 5–8 scored mild depression; 9–11 moderate depression; and 12–15 severe depression [30]. The validity and reliability of the study instruments were checked as the instruments were used in the Bengali version. To measure the reliability the value of Cronbach alpha was found as 0.901 which suggests a high degree of internal consistency and the instruments were found significantly valid at 95% CI. To determine the validity we compared the obtained reliability score with the instruments of other studies in Bangladesh and India [18,29]. Study instruments were peer-reviewed by the authors and experts in the relevant field. In addition, the validity was tested through Pearson’s Correlation Coefficient analysis. Considering the degree of freedom at the two-tailed test as (N-2 = 200-2 = 198) at 95% Confidence Interval (CI) the obtained value (0.330) was found greater than the critical tabulated value (0.138). The outcome suggests the significant validity of the instrument.

A binomial logistic regression analysis was performed followed by a modeling procedure considering a backward elimination process, including pre-specified confounders i.e. age, gender, education, past occupation, co-morbidities, physical disability for regular work, and reasons for staying at the old home. Odds ratios with 95% confidence intervals concerning cognitive impairment status and depression status were calculated separately for the specified exposures.

## 3 Results

### 3.1 Participant’s characteristics

A total of 200 respondents were recruited in this study with 50.5% male and a mean (±SD) age of 68.13 (±5.83) years. More than half of the respondents (64%, n = 128/200) were aged ≤70 years, and majorities (76%, n = 152/200) of the respondents were married. In addition, about half of the study subjects (44.5%, n = 89/200) had a Higher Secondary Certificate (HSC) or above education, while 36% did not have any formal education. Moreover, the study revealed that nearly half of the study subjects (47%, n = 94/200) were service holders as their past occupation. Unexpectedly study revealed a depressive scenario in that most of them were staying at old homes due to their children’s decisions (56%, n = 112/200). (Table 1).

**Table 1:** Characteristics of the respondents determining their Cognitive Impairment status (n = 200).

Characteristics	Number of participants, n (%)	Level of Cognitive Impairment		
		Normal, n (%)	Mild/Moderate/Severe, n (%)	ꭕ^2^/p-value (≤0.05)
**Age Group (In Years)**				
≤70	128 (64)	19 (9.5)	109 (54.5)	7.36/0.03*
>70	72 (36)	20 (10)	52 (26)	
**Gender**				
Male	101 (50.5)	29 (14.5)	72 (36)	38.12/0.01*
Female	99 (49.5)	10 (5)	89 (44.5)	
**Education**				
Non-formal Education	72 (36)	2 (1)	70 (35)	91.82/0.01*
≤Primary	15 (7.5)	1 (0.5)	14 (7)	
≤Secondary	24 (12)	3 (1.5)	21 (10.5)	
≥Higher Secondary	89 (44.5)	33 (16.5)	56 (28)	
**Past Occupation**				
Service holder	94 (47)	32 (16)	62 (31)	74.26/0.01*
Housewife	80 (40)	5 (2.5)	75 (37.5)	
Businessman	26 (13)	2 (1)	24 (12)	
**Reasons for Staying at Old Home**				
Self-desire	66 (33)	25 (12.5)	41 (20.5)	75.26/0.01*
Children Choice	112 (56)	5 (2.5)	107 (53.5)	
No shelter/Others	22 (11)	9 (4.5)	13 (6.5)	
**Difficulties with Manipulation (Physical Disability)**				
Yes	18 (9.3)	8 (4.1)	10 (5.2)	19.39/0.01*
No	175 (90.7)	30 (15.5)	145 (75.1)	

Data are presented as frequency (n), percentage (%); *Statistical significance at p-value ≤ 0.05. Chi-square test was used to observe the association.

### 3.2 Collective mental health status of the senior citizens

**Fig. 1:**
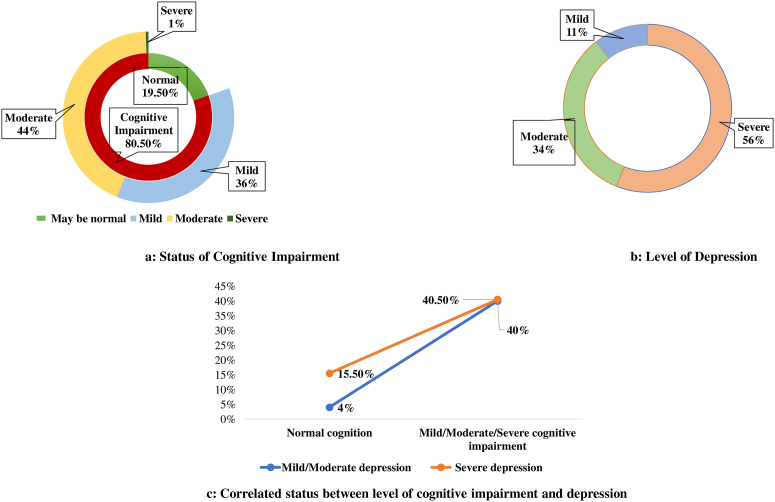
Collective mental health status of the study subjects (n = 200).

As collective mental health status, this study assesses the level of cognitive impairment and depression among the study subjects. Among all the study subjects, majorities (80.50%, n = 161/200) were found as impaired (Moderate: 44%, mild: 36%, and severe: 1%) in their cognitive status in contrast to the others (19.50%) with normal cognition (Fig. 1a).

More than half of the senior citizens (56%, n = 112/200) were found as severely depressed where a significant amount of 33.5% (n = 67/200) had moderate depression and only 11% had mild depression (Fig. 1b).

The study revealed a significant association between the cognitive impairment status and the depression status of the respondents. It is observed that subjects who had any form of cognitive impairment they found more likely to be depressed compared to others (ꭕ2/p = 10.85/0.01& COR/p = 3.83/0.01; 95% CI: 1.66–8.83) (Fig. 1c).

### 3.3 Respondent’s characteristics associated with their cognitive impairment status

Results of bivariate (cross table) analysis revealed that respondent’s age (p = 0.03), gender (p = 0.01), education (p = 0.01), past occupation (p = 0.01), difficulties with manipulation as physical disability due to aging (p = 0.01), and children’s choice as a reason for staying at old home (p = 0.01) is significantly associated with their status of cognitive impairment. In the other words, the study also observed that any form of cognitive impairment is significant among the females and the subjects who were aged ≤70 years in comparison to their counters whereas the group of non-formal educated subjects was found as the first and HSC/higher educated as the second largest group having any form of cognitive impairment. In addition, cognitive impairment was predominantly found among the senior citizens who were housewives and service holders as their past occupation. Furthermore, respondents, who had significant cognitive impairment were staying at old homes according to the decision of their children. This scenario indicates that such a health problem seems to be a severe burden for the family members which is why cases with cognitive impairment do not get any support from their peers in the family (Table 1).

### 3.4 Identified predictors triggering cognitive impairment status among the respondents

This study revealed significant predictors associated with the cognitive impairment status among the respondents as the vital outcome of regression analysis. Significant variables from cross-tabulation were enrolled in the regression analysis procedure. Female respondents belonging less than or equal to 70 years of age were found more significant to having cognitive impairment rather than the counter age group (≤70 years: COR/p = 2.21/0.03; 95% CI: 1.09–4.49, female: COR/p = 3.58/0.01; 95% CI: 1.64–7.84). In addition, respondents who were housewives and businessmen were found to have significant cognitive impairment compared to the service holder group (housewife: COR/p = 7.74/0.01; 95% CI: 2.85–21.06, businessman: COR/p = 6.19/0.02; 95% CI: 1.38–27.88) and concerning the education as the predictor, significant higher odds of cognitive impairment found among the respondents with lower educational status like non-formal (COR/p = 20.63/0.01; 95% CI: 4.74–89.69), up to primary (COR/p = 8.25/0.05; 95% CI: 1.04–65.63), and up to secondary (COR/p = 4.13/0.03; 95% CI: 1.14–14.89). Exploring the reasons behind the decision of staying at an old home, the study significantly observed a miserable scenario which interprets as respondents with cognitive impairment (COR/p = 14.82/0.05; 95%CI: 4.31–50.97) were staying at an old home according to their children’s choice.

**Fig. 2:**
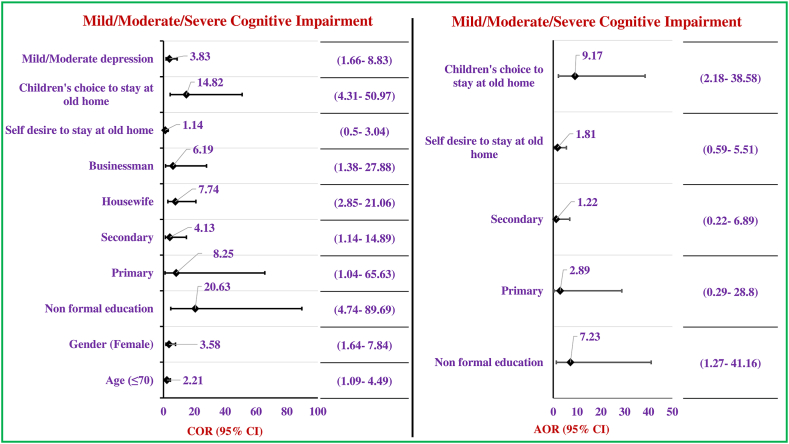
Predictors influencing the cognitive impairment status of the respondents (n = 200). Fig. 2 footnote: Statistically significant predictor is considered at p ≤ 0.05. The reference category for gender is male, education is HSC/above, for past occupation is service holder, and lastly for reasons of staying old home is no shelter/others. (Regression Analysis).

The study revealed the final predictors triggering the status of cognitive impairment after the adjustment regression modeling with the significant independent variables of crude odds and elimination of the confounders in a backward manner. Here, mild/moderate/severe cognitive impairment is explored as a dependent variable against the normal cognition respondents. After all, it was identified that non-formal educated respondents (AOR/p = 7.23/0.03; 95% CI: 1.27–41.16) who did not have any family care support were found to have a significant cognitive impairment which made their fate to stay at old home upon their children’s choice (AOR/p = 9.17/0.01; 95% CI: 2.18–38.58) (Fig. 2).

### 3.5 Respondent’s characteristics associated with their status of depression

This study also revealed some crucial determinants for the depression status among the study subjects using another bivariate (cross table) analysis procedure. The outcome revealed as respondent’s age (p = 0.01), gender (p = 0.01), education (p = 0.01), past occupation (p = 0.01), having the cardiovascular disorder as comorbidities (p = 0.05), and children’s choice as a reason for staying at old home (p = 0.01) are significantly associated with their level of depression. Figuring out all the demographic factors that may trigger one’s state of depression, the study also observed that any form of severe depression is significant among females aged ≤70 years in comparison to their counters by way of explanation. More intense depression was also significantly found among the higher (more than or equal higher secondary) educated subjects. Due to a lack of self-motivation subjects with lower educational backgrounds were more prone to severe depression, the other subjects with a higher secondary degree and above were found to have mild to moderate depression. In addition, senior citizens who were service holders in their past occupations were suffering from mild depression whereas those who were housewives in their lifetime got severe depression. Furthermore, respondents who were staying at old homes according to their self-desire and did not have any shelter were found more depressed rather than the counter group. As a whole, it is reflected that educated senior citizens got more depressed due to their miserable status as a result of not having any shelter or staying at the old home on their self-desire although having family members in some cases (Table 2).

**Table 2:** Characteristics determining the depression level of the respondents (n = 200).

Characteristics	Number of participants, n (%)	Level of Depression		
		Mild/Moderate, n (%)	Severe, n (%)	ꭕ^2^/p-value (≤0.05)
**Age Group (In Years)**				
≤70	128 (64)	62 (31)	66 (33)	8.25/0.01*
>70	72 (36)	50 (25)	22 (11)	
**Gender**				
Male	101 (50.5)	70 (35)	31 (15.5)	14.66/0.01*
Female	99 (49.5)	42 (21)	57 (28.5)	
**Education**				
Non-Formal Education	72 (36)	22 (11)	50 (25)	54.03/0.01*
≤Primary	15 (7.5)	4 (2)	11 (5.5)	
≤Secondary	24 (12)	11 (5.5)	13 (6.5)	
≥Higher Secondary	89 (44.5)	75 (37.5)	14 (7)	
**Past Occupation**				
Service holder	94 (47)	76 (38)	18 (9)	44.60/0.01*
Housewife	80 (40)	28 (14)	52 (26)	
Businessman	26 (13)	8 (4)	18 (9)	
**Reasons for Staying at Old Home**				
Children Choice	112 (56)	69 (34.5)	43 (21.5)	32.41/0.01*
No shelter/Others	22 (11)	6 (3)	16 (8)	
Self-desire	66 (33)	13 (6.5)	53 (26.5)	
**Comorbidities (Cardiac Disorder)**				
Yes	18 (9)	14 (7)	4 (2)	3.81/0.05*
No	182 (91)	98 (49)	84 (42)	

Data are presented as frequency (n), percentage (%); *Statistical significance at p-value ≤0.05. Chi-square test was used to observe the association.

### 3.6 Identified predictors influencing the status of depression among the respondents

This study also revealed some significant predictors associated with the depression status among the respondents by performing regression analysis again. Significant variables from the bivariate analysis were included in the regression analysis. Surprisingly, male respondents who were aged less than or equal to 70 years were found to have severe depression rather than the counter age group (≤70 years: COR/p = 2.42/0.01; 95% CI: 1.32–4.45, male: COR/p = 3.07/0.01; 95% CI: 1.71–5.48). Besides that, unfortunately, it was observed that respondents who had higher education (HSC or above: COR/p = 12.18/0.01; 95% CI: 5.70–26.03) were found to be severely depressed compared to those with primary and secondary level educational backgrounds. Considering occupation as the predictor, significantly higher odds for severe depression was found among the respondents who were service holder (COR/p = 9.5/0.01; 95% CI: 3.57–25.28) in their previous occupation. Finally, exploring the reasons behind staying at the old home, this study surprisingly observed that, their own decision (COR/p = 4.28/0.01; 95% CI: 1.55–11.78) and not having any shelter (COR/p = 6.54/0.01; 95% CI: 3.19–39.39) were the significant dominating reasons and this finding indicates, the background for the severe depression at this stage from the real emotional point of view.

**Fig. 3:**
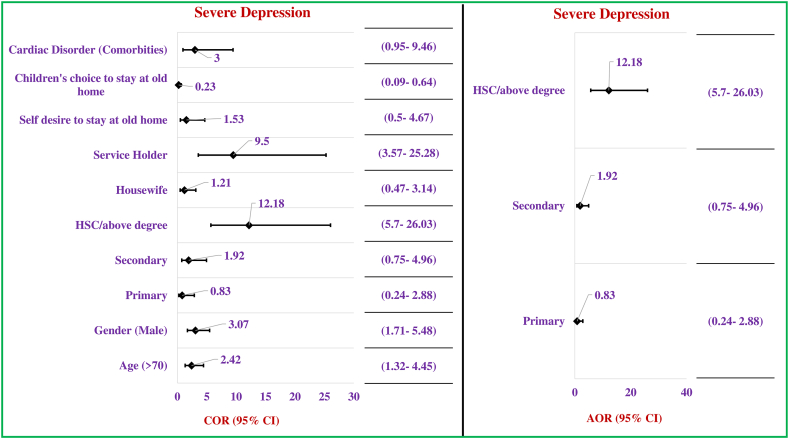
Predictors influencing the depression level of the respondents (n = 200). Fig. 3 footnote: Statistically significant predictor is considered at p ≤ 0.05. The reference category for gender is male, education is HSC/above, for past occupation is service holder, for cardiac disorder is no cardiac disorder and lastly for reasons of staying old home is children’s choice. (Regression Analysis).

Final predictors were identified after the model fitness including the significant variables with crude odds and eliminated all the confounders in a backward manner which accelerate the status of depression severely. Here, severe depression is explored against mid/moderate depression status. Regression modeling for Cognitive impairment and depression status was done separately. Finally, respondents’ education was found significant to trigger their state of depression in severe conditions. By way of explanation, higher educated respondents (AOR/p = 12.18/0.01; 95% CI: 5.69–26.03) were found to suffer from severe depression as they could not accept the fate of staying at an old home for their care and support (Fig. 3).

## 4 Discussion

Cognitive impairment and depression among older adult people vary across different setups such as old age homes (OAHs), communities, medical clinics, etc. Depression is one of the most common psychiatric disorders among old people which may trigger cognitive impairment among them.

This study mainly focused on accessing the impact of significant factors & predictors on cognitive impairment as well as the depression status of the older residents in the old home. In the current context of our country, it is crucial to improve the elderly care support system considering their good mental health which might reduce the additional health-related social and economic burdens of the country.

The cognitive status of the respondents revealed that majorities (80.50%) were found as impaired in their cognitive function. Among them, 44% had moderate and 36% had mild cognitive impairment while only 1% were found severely impaired in their cognition. Findings from other international studies among the community-based (India) and elderly care home (India and Malaysia) based old-aged groups support the scenario of this current study [2,31]. A similar prevalence of dementia (36.7%) was found among older adults in nursing homes in China [32]. These resemblances might be due to almost similar socio-demographic characteristics along with attitudes towards older people including cultural factors which is alarming. In Bangladesh, cognitive impairment was found at 27.9% among older adults in a community-based cross-sectional study [9]. However, this critical issue is not explored among Bangladeshi old home residents.

On the other hand, this study explored more than half (56%) of the senior citizens as severely depressed where a significant amount 33.5% had moderate depression and only 11% had mild depression. A previous cross-sectional survey in Bangladesh robust a similar prevalence (55.5%) of depression among the older adults of the Meherpur district [20]. While the portrayal of depression among the elderly home care population in Bangladesh showed that 84% had depressive symptoms [19]. Another study from Rajkot shows that the depressive status of the residents in old age homes is much higher (46%) rather than community (33%) people [15]. These pictures are mostly similar in this Indian sub-continent due to unacceptable negligence from the family members in most of the cases.

Cognitive impairment was found significantly associated with the level of depression among the respondents. It is observed that subjects who have any form of cognitive impairment they found more likely to be depressed compared to others (χ2/p = 10.85/0.01& OR/p = 3.83/0.01). A study by JAMA psychiatry shows that depression is very much common in individuals with mild cognitive impairment (MCI) which was 32% (95% CI, 27–37), and the prevalence of depression with MCI significantly (p < 0.001) differs between community & clinic-based sample [33]. This scenario indicates that subjects with cognitive impairment feel depressed due to their miserable situation. Intellectual shreds of evidence from Bangladesh explored both issues separately concerning the nutritional status, social activities, cultures, any other dependencies, etc [9,10,19,20,34]. For proper screening and minimizing the consequences of cognitive impairment, it is pivotal to explore the extent of influence of depressive symptoms toward the development of dementia among older adults living in old homes in Bangladesh. This strategy may increase the sustainable health and well-being of neglected older people living in old homes.

Female respondents who were less than or equal to 70 years of age were found more significant to having cognitive impairment rather than the counter age group (≤70 years: COR/p = 2.21/0.03; 95% CI: 1.09–4.49, female: COR/p = 3.58/0.01; 95% CI: 1.64–7.84). In addition, respondents who were housewives and businessmen were found to have significant cognitive impairment compared to the service holder group. This is mostly due to a lack of social engagement and less activity of elder women in the context of our country. A study from Bangladesh shows that the prevalence of cognitive impairment is very much alarming and it is much higher among older adult women. Age (odds ratio, COR = 1.06, p = 0.046, 95% CI: 1.001 to 1.119) and social engagement (OR = 0.25, p = 0.033, 95% CI: 0.072 to 0.898) were found to be statistically significant predictors of cognitive impairment among the older women as well as the scenario of more socially activity resulting in less cognitive decline in old age [9,34]. Study findings indicate that active participation in every aspect of life’s journey can be supportive to prevent cognitive impairment as well as depression. Moreover, cognitively impaired subjects were staying at the old homes of their children’s choice. This scenario indicates that such a health problem seems to be a severe burden for the family members which is why cases they do not get any support from their peers in the family and as a consequence, they need to move into an old home.

In the case of depression status, it was found that male respondents who were less than or equal to 70 years were found to have severe depression rather than the counter age group. Other studies in Bangladesh robust this outcome also adding the nutritional status, type of residence, and comorbidities as significant risk factors for depression [19,20]. Educational level was found significantly (p < 0.01) associated with severe depression. A contrasting picture was found in a study of Egypt as there is no significant association (p < 0.582) between educational level & depression [24]. This contrasting finding reflects the socio-demographic & cultural variances of Bangladesh with Egypt. As an emotional nation highly educated Bangladeshi subjects were found to be more depressed due to their miserable status of not having any shelter or staying at the old home on their self-desire although having family members in some cases. Maintaining a good physical state, a healthy lifestyle, and a supportive family environment can reduce depression in older adults suggested by other studies [19,20,24].

Depressed old people who are living alone have a greater risk to develop cognitive impairment & cognitive function is known to be associated with depression. Some scenarios of this study revealed several triggering factors, which lead the older subjects to be functionally disabled with increasing the level of depression. To evaluate the reasons for staying at the old home of the study subjects, the study significantly observed a miserable picture where respondents with cognitive impairment were staying at the old home according to their children’s choice. Most of the studies from Asia support our finding with very few contrast pictures too [2,18,24,27].

As the solution of heading towards the improvement of the mental health of older adults, especially in old homes, caregivers, family members, and older adults need to be educated about mental health and well-being. Frequent dutiful contact with family members is also recommended for the psychological well-being of this deprived group including scaling up holistic healthcare support through multisectoral approaches and strategic policy development. Likewise, Rahman et al. and Mazumder et al. in their studies recommended such effective strategies after exploring the unmet mental need and mental health status of older adults in Bangladesh. Still, mental health professionals are providing various psychological aids as tips through social media which might bring a fruitful outcome if complied properly by the family members and also caregivers or health care providers in old homes [35,36]. However, in Bangladesh the poor mental health status of older adults in old homes is prevalent and it is pivotal to do more empirical studies on this vulnerable group which lacks miserably. This study tried to explore the collective mental health status with the determinants of this group. This endeavor might be unique guidance for more insightful explorations in the future to fix the mental health issues among older adults in old homes in Bangladesh and improve their health status.

As a limitation, the samples were taken from the old homes of Dhaka city only which may differ from the original nationwide scenario. The results do not represent the entire population of Bangladesh. Eligible sample selection was difficult due to their unstable physical and mental condition in their overaged situation which paused delining response rate during sample size determination. Besides, it was a cross-sectional study, and causality cannot be determined. Finally, to explain the strength, this study explored both cognitive impairment and depression status simultaneously in the same respondents which is a unique investigation of this group in Bangladesh. Duel burden of these two prevalent mental health issues may pose a critical risk of severe health issues in society which increase the economic burden too. In addition, this study sorted out some crucial predictors which will be helpful for the policymakers in rethinking new dimensions among the residents of elderly care homes. Our study may also help to introduce a different pattern of intervention on a large scale for reducing depressive states with functional disabilities as well as cognitive impairment among older people. The unique outcome of this study may guide policymakers in scaling up special healthcare strategies in existing healthcare facilities for the well-being of older adults in Bangladesh.

## 5 Conclusion

To summarize, the study revealed a miserable scenario regarding cognitive impairment and depression among old people who are living in old homes in Bangladesh. Nearly half of the respondents were found to have moderate cognitive impairment whereas more than half of the respondents had severe depression. In addition, significant severe depression was found among the higher educated respondents as they were aware of their negative status in life. Furthermore, it was found that cognitive impairment is strongly related to severe depression. Functional disabilities along with cognitive impairment of the older adults were found to be the major concerns behind the decision for living in geriatric homecare in Bangladesh. Therefore, study findings finally recommend that nationwide intervention programs or measures are needed to combat the situation of this sensitive issue of geriatric people. The outcome of this study suggests that reassurance of family support is mandatory and also rethinking policymakers about the well-being of older adults, especially from old homes. Further, population-based and interventional studies are suggested to convert such burdened populations into competent resources through make them active as well as improving their cognitive and depression status.

## Source of funding

This research did not receive any specific grant from funding agencies in the public, commercial, or not-for-profit sectors.

## Author contribution statement

Nasrin Akter: Conceived and designed the study; performed the study; analyzed and interpreted the data; contributed materials, analysis tools, or data; wrote the paper.

Bilkis Banu: Conceived and designed the study; performed the study; analyzed and interpreted the data; contributed materials, analysis tools, or data; wrote the paper.

Sujana Haque Chowdhury: Performed the study; analyzed and interpreted the data; contributed materials, analysis tools, or data; wrote the paper.

Kazi Rakibul Islam: Performed the study; analyzed and interpreted the data; contributed materials, analysis tools, or data; wrote the paper.

Tahsin Tasneem Tabassum: Performed the study; contributed materials, analysis tools, or data; wrote the paper.

Sarder Mahmud Hossain: Performed the study; contributed materials, analysis tools, or data; wrote the paper.

## Data availability statement

Data included in article/supp. material/referenced in article.

## Declaration of competing interest

The authors declare that they have no known competing financial interests or personal relationships that could have appeared to influence the work reported in this paper

## References

[1] Ageing population in Bangladesh (2022). https://ageingasia.org/ageing-population-bangladesh/#:%7E:text=As%20of%202019%2C%20over%2013,will%20be%20a%20senior%20citizenR.

[2] Samuel R., McLachlan C.S., Mahadevan U., Isaac V. (2016). Cognitive impairment and reduced quality of life among old-age groups in Southern Urban India: home-based community residents, free and paid old-age home residents. QJM: Int. J. Med..

[3] Ferreira A.R., Dias C.C., Fernandes L. (2016). Needs in nursing homes and their relation with cognitive and functional decline, behavioral and psychological symptoms. Front. Aging Neurosci..

[4] Seifaddini R., Tajadini H., Choopani R. (2015). Physiopathology of dementia from the perspective of traditional Persian medicine. J. Evid. Based Complementary Altern. Med..

[5] Nejati V. (2010). Cognitive-executive functions of brain frontal lobe in aged adults. Int. J. Behav. Sci..

[6] Caracciolo B., Palmer K., Monastero R., Winblad B., Bäckman L., Fratiglioni L. (2008). Occurrence of cognitive impairment and dementia in the community: a 9-year-long prospective study. Neurology.

[7] Paúl C., Ribeiro O., Santos P. (2010). Cognitive impairment in old people living in the community. Arch. Gerontol. Geriatr..

[8] Unverzagt F.W. (2011). Incidence and risk factors for cognitive impairment no dementia and mild cognitive impairment in African Americans. Alzheimer Dis. Assoc. Disord..

[9] Hossain Z., Das J.K., Fahim N.F., Ali H., Khondoker M. (2021). Prevalence and predictors of cognitive impairment among the elderly in Bangladesh. J. Public Health.

[10] Uddin M.S., Mamun A.A., Takeda S., Sarwar M.S., Begum M.M. (2019). Analyzing the chance of developing dementia among geriatric people: a cross‐sectional pilot study in Bangladesh. Psychogeriatrics.

[11] Sosa A.L. (2012). Prevalence, distribution, and impact of mild cognitive impairment in Latin America, China, and India: a 10/66 population-based study. PLoS Med..

[12] Tiwari S.C., Pandey N.M., Singh I. (2012). Mental health problems among inhabitants of old age homes: a preliminary study. Indian J. Psychiatr..

[13] Luk J.K., Chan W.K., Ng W.C., Chiu P.K., Ho C., Chan T.C., Chan F.H. (2013). Mortality and health services utilisation among older people with advanced cognitive impairment living in residential care homes. Hong Kong Med. J..

[14] Feng L., Yap K.B., Ng T.P. (2013). Depressive symptoms in older adults with chronic kidney disease: mortality, quality of life outcomes, and correlates. Am. J. Geriatr. Psychiatr..

[15] Ho C.S., Feng L., Fam J., Mahendran R., Kua E.H., Ng T.P. (2014). Coexisting medical comorbidity and depression: multiplicative effects on health outcomes in older adults. Int. Psychogeriatr..

[16] Banerjee A., Kumar S., Kulhara P., Gupta A. (2008). Prevalence of depression and its effect on disability in patients with age-related macular degeneration. Indian J. Ophthalmol..

[17] Ghaderi S., Sahaf R., Mohammadi Shahbalaghi F., Ansari G., Gharanjic A., Ashrafi K. (2012). Prevalence of depression in elderly Kurdish community residing in Boukan, Iran. Iran. J. Ageing.

[18] Zalavadiya D.D. (2017). A comparative study of depression and associated risk factors among elderly inmates of old age homes and community of Rajkot: a Gujarati version of the geriatric depression scale-short form (GDS-G). Indian J. Community Med..

[19] Alam M.R. (2021). Geriatric malnutrition and depression: evidence from elderly home care population in Bangladesh. Prev. Med. Rep..

[20] Rahman M.S. (2020). Determinants of depressive symptoms among older people in Bangladesh. J. Affect. Disord..

[21] Wahlin Å., Palmer K., Sternäng O., Hamadani J.D., Kabir Z.N. (2015). Prevalence of depressive symptoms and suicidal thoughts among elderly persons in rural Bangladesh. Int. Psychogeriatr..

[22] Disu T.R., Anne N.J., Griffiths M.D., Mamun M.A. (2019). Risk factors of geriatric depression among elderly Bangladeshi people: a pilot interview study. Asian J. Psychiatry.

[23] Pilania M., Bairwa M., Kumar N., Khanna P., Kurana H. (2013). Elderly depression in India: an emerging public health challenge. Australas. Med. J..

[24] Kumar S., Joseph S., Abraham A. (2021). Prevalence of depression amongst the Elderly population in old age homes of Mangalore city. J. Fam. Med. Prim. Care.

[25] Mehta K.M., Yaffe K., Covinsky K.E. (2002). Cognitive impairment, depressive symptoms, and functional decline in older people. J. Am. Geriatr. Soc..

[26] Borhaninezhad V.R., Kazazi L., Haghi M., Chehrehnegar N. (2016). Quality of life and its related factors among elderly with diabetes (Per-sian). Iran. J. Ageing.

[27] Khan M.R.K., Rizvi A.N., Habib M.A., Hasan M.K., Mamun A., Alam M.R., Islam R. (2016). Etiological pattern of dementia in Bangladesh. Bangladesh J. Neurosci..

[28] Moral S. (2022). https://en.prothomalo.com/bangladesh/49ozhgo002.

[29] Ghose S.K., Ahmed K.G.U., Chowdhury A.H., Hasan A.H., Khan M.Z.R., Karim A.R. (2016). Adapting Bangla mini-mental state examination (MMSE-B) among healthy elderly in Bangladesh. Bangladesh J. Neurosci..

[30] Greenberg S.A. (2012). The geriatric depression scale (GDS). Best Pract. Nurs. Care to Older Adults.

[31] Al-Jawad M., Rashid A.K., Narayan K.A. (2007). Prevalence of undetected cognitive impairment and depression in residents of an elderly care home. Med. J. Malaysia.

[32] Guo M. (2012). Prevalence of dementia and mild cognitive impairment in the elderly living in nursing and veteran care homes in Xi’an, China. J. Neurol. Sci..

[33] Ismail Z. (2017). Prevalence of depression in patients with mild cognitive impairment: a systematic review and meta-analysis. JAMA Psychiatry.

[34] James B.D., Wilson R.S., Barnes L.L., Bennett D.A. (2011). Late-life social activity and cognitive decline in old age. J. Int. Neuropsychol. Soc..

[35] Rahman M.S., Rahman M.A., Afroze L., Islam S.M.S. (2020). Unmet needs for mental care services for older people in Bangladesh during the COVID-19 pandemic. Gen. Psychiatry.

[36] Mazumder H., Murshid M.E., Faizah F., Hossain M.M. (2020). Geriatric mental health in Bangladesh: a call for action. Int. Psychogeriatry.

