# Supplementary material for: Astute exploration of collective mental health events among the residents of elderly care homes
Source: Heliyon. 2023 Jul 8;9(7):e18117. doi: 10.1016/j.heliyon.2023.e18117 (PMC10366419; doi:10.1016/j.heliyon.2023.e18117)
Supplement: Multimedia component 1 [file mmc1.doc]

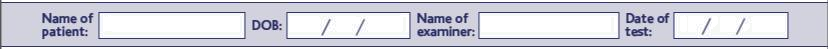


**Standardised Mini-Mental State Examination (SMMSE)**

***Please see accompanying guidelines for administration and scoring instructions***

**Say:** *I am going to ask you some questions and give you some problems to solve. Please try to answer as* *best you can.*

**1.** Allow ten seconds for each reply. **Say:**

| a) *What year is this?* (accept exact answer only) | /1 |
| --- | --- |
| b) *What season is this?* (during the last week of the old season or first week of a new |  |
| season, accept either) | /1 |
| c) *What month is this?* (on the first day of a new month or the last day of the previous |  |
| month, accept either) | /1 |
| d) *What is today’s date?* (accept previous or next date) | /1 |
| e) *What day of the week is this?* (accept exact answer only) | /1 |

**2.** Allow ten seconds for each reply. **Say:**

| a) *What country are we in?* (accept exact answer only) | /1 |
| --- | --- |
| b) *What state are we in?* (accept exact answer only) | /1 |
| c) *What city/town are we in?* (accept exact answer only) | /1 |

1. <At home> *What is the street address of this house?* (accept street name and house

| number or equivalent in rural areas) | /1 |
| --- | --- |

<In facility> *What is the name of this building?* (accept exact name of institution only) /1

| e) <At home> *What room are we in?* (accept exact answer only) | /1 |
| --- | --- |
| <In facility> *What floor of the building are we on?* (accept exact answer only) | /1 |

1. **Say:** *I am going to name three objects. When I am finished, I want you to repeat them. Remember* *what they are because I am going to ask you to name them again in a few minutes* (say slowly at approximately one-second intervals).

**Ball Car Man**

For repeated use: Bell, jar, fan; bill, tar, can; bull, bar, pan

**Say:** *Please repeat the three items for me* (score one point for each correct reply on the first

attempt) /3

Allow 20 seconds for reply; if the person did not repeat all three, repeat until they are learned or up to a maximum of five times (but only score first attempt)

1. **Say:** *Spell the word WORLD* (you may help the person to spell the word correctly). **Say:** *Now spell it* *backwards please* (allow 30 seconds; if the person cannot spell world even with assistance, score zero). Refer to accompanying guide for scoring instructions (score on reverse of this sheet)

|  |  | /5 |
| --- | --- | --- |
| **5.** | **Say:** *Now what were the three objects I asked you to remember?* | /3 |
|  | (score one point for each correct answer regardless of order; allow ten seconds) |  |
| **6.** | Show wristwatch. **Ask:** *What is this called?* | /1 |

(score one point for correct response; accept ‘wristwatch’ or ‘watch’; do not accept ‘clock’ or ‘time’, etc.; allow ten seconds)

1

| **7. Show pencil. Ask:** *What is this called?* | /1 |
| --- | --- |

(score one point for correct response; accept ‘pencil’ only; score zero for pen; allow ten seconds for reply)

| **8. Say:** *I would like you to repeat a phrase after me: No ifs, ands, or buts* | /1 |
| --- | --- |

(allow ten seconds for response. Score one point for a correct repetition. Must be exact, e.g. no ifs or buts, score zero)

| **9. Say:** *Read the words on this page and then do what it says* | /1 |
| --- | --- |

Then, **hand** the person the sheet with CLOSE YOUR EYES (score on reverse of this sheet) on it. If the subject just reads and does not close eyes, you may repeat: *Read the words on this page and* *then do what it says*, a maximum of three times. See point number three in Directions for Administration section of accompanying guidelines. Allow ten seconds; score one point only if the person closes their eyes. The person does not have to read aloud.

1. **Hand** the person a pencil and paper. **Say:** *Write any complete sentence on that piece of paper*

| (allow 30 seconds. Score one point. The sentence must make sense. Ignore spelling errors). | /1 |
| --- | --- |

1. **Place** design (see page 3), pencil, eraser and paper in front of the person. **Say:** *Copy this design*

| *please.* Allow multiple tries. | /1 |
| --- | --- |

Wait until the person is finished and hands it back. Score one point for a correctly copied diagram. The person must have drawn a four-sided figure between two five-sided figures. Maximum time: one minute.

1. **Ask** the person if he is right or left handed. **Take** a piece of paper, hold it up in front of the person and **say** the following: *Take this paper in your right/left hand* (whichever is non-dominant), *fold the* *paper in half once with both hands and put the paper down on the floor*.

| Takes paper in correct hand_________ | /1 |
| --- | --- |
| Folds it in half___________ | /1 |
| Puts it on the floor________ | /1 |
| **TOTAL TEST SCORE:** | **/30** |
| ADJUSTED SCORE: | / |

*The SMMSE tool and guidelines are provided for use in Australia by the Independent Hospital Pricing Authority under a licence agreement with the copyright owner, Dr D. William Molloy. The SMMSE Guidelines for administration and scoring instructions and the SMMSE tool must not be used outside Australia without the written consent of*

*Dr D. William Molloy.*

Molloy DW, Alemayehu E, Roberts R. Reliability of a standardized Mini-Mental State Examination compared with the traditional Mini-Mental state Examination. *American Journal of Psychiatry*, Vol. 14, 1991a, pp.102-105.

2


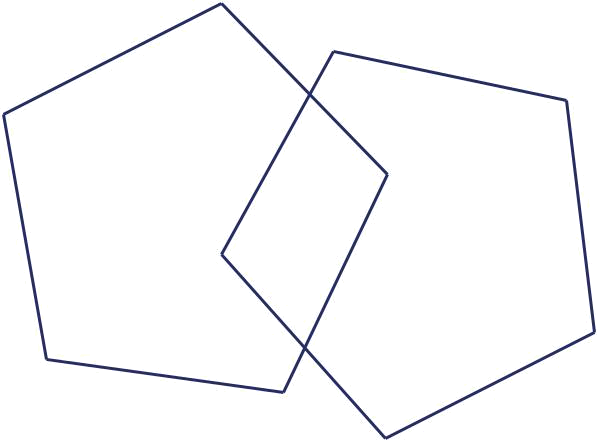


Time:

----------------------------------------------------------------------------------------------------------------------------------------------


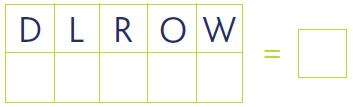


----------------------------------------------------------------------------------------------------------------------------------------------

CLOSE YOUR EYES

3
