# Supplementary material for: Astute exploration of collective mental health events among the residents of elderly care homes
Source: Heliyon. 2023 Jul 8;9(7):e18117. doi: 10.1016/j.heliyon.2023.e18117 (PMC10366419; doi:10.1016/j.heliyon.2023.e18117)
Supplement: Multimedia component 2 [file mmc2.docx]

**Geriatric Depression Scale**

Circle the answer that best describes how you felt over the past week.

1. Are you basically satisfied with your life? yes no

2. Have you dropped many of your activities and interests? yes no

3. Do you feel that your life is empty? yes no

4. Do you often get bored? yes no

5. Are you in good spirits most of the time? yes no

6. Are you afraid that something bad is going to happen to you? yes no

7. Do you feel happy most of the time? yes no

8. Do you often feel helpless? yes no

9. Do you prefer to stay at home, rather than going out and doing things? yes no

10. Do you feel that you have more problems with memory than most? yes no

11. Do you think it is wonderful to be alive now? yes no

12. Do you feel worthless the way you are now? yes no

13. Do you feel full of energy? yes no

14. Do you feel that your situation is hopeless? yes no

15. Do you think that most people are better off than you are? yes no

Total Score……………

**Instructions:** Score 1 point for each bolded answer. A score of 5 or more suggests depression.

1. Are you basically satisfied with your life? yes **no**

2. Have you dropped many of your activities and interests? **yes** no

3. Do you feel that your life is empty? **yes** no

4. Do you often get bored? **yes**  no

5. Are you in good spirits most of the time? yes **no**

6. Are you afraid that something bad is going to happen to you? **yes**  no

7. Do you feel happy most of the time? yes **no**

8. Do you often feel helpless? **yes** no

9. Do you prefer to stay at home, rather than going out and doing things? **yes**  no

10. Do you feel that you have more problems with memory than most? **yes**  no

11. Do you think it is wonderful to be alive now? yes **no**

12. Do you feel worthless the way you are now? **yes**  no

13. Do you feel full of energy? yes **no**

14. Do you feel that your situation is hopeless? **yes**  no

15. Do you think that most people are better off than you are? **yes**  no

A score of > 5 suggests depression Total Score
